# MiRs in RA: possible biomarkers and therapeutic targets

**DOI:** 10.1186/ar3615

**Published:** 2012-02-09

**Authors:** Maria Filkova, Caroline Ospelt, Joanna Stanczyk, Serena Vettori, Ladislav Senolt, Mojca Frank, Christoph Kolling, Beat A Michel, Renate E Gay, Steffen Gay, Astrid Jüngel

**Affiliations:** 1 Center of Experimental Rheumatology, University Hospital Zurich, Zurich, Switzerland; 2 Institute of Rheumatology, Department of Experimental Rheumatology of the 1st Faculty of Medicine, Charles University in Prague, Prague, Czech Republic; 3 Schultess Clinic, Zurich, Switzerland

## Background and objective

New concepts of therapy highlight an early use of effective treatment to prevent further joint damage in RA. Altered expression of epigenetic marks like miRs offers us the possibility to develop new diagnostic tools and novel therapeutic targets.

We found miR-146, -155 and -203 to be upregulated in rheumatoid arthritis (RA) synovial fibroblasts (SF) compared to osteoarthritis (OA) SF [1,2]. Based on the comprehensive analysis of the expression of 260 miRs we found miR-196a to be one of the most downregulated miRs in RASF. In peripheral blood mononuclear cells, miR-132 and -223 are upregulated in established RA compared with healthy controls (HC) [3,4].

Our aim was to analyze miRs as potential systemic markers in early stages of the disease and to find new miRs locally at the site of inflammation that play a role in the pathogenesis of RA.

## Methods

MiRs from sera of patients with treatment naïve early RA (ERA), with treated established RA and HC were isolated by phenol-chloroform extraction. TaqMan Low Density Array was used to analyze the expression of 260 miRs in RASF and OASF. MiR-196a expression was further analyzed in additional RASF and OASF, RA and OA synovial tissues. TaqMan RealTime-PCR was used for quantification of miRs and functional experiments (MTT, scratch assay, AnnexinV FACS) were performed following transfection with pre-miR or miR-196a inhibitor.

## Results

In sera of patients with ERA, the expression of miR-146a was lower than in both HC (p < 0.05) and established RA sera (p < 0.001) while miR-155, 132, -203 and -223 showed no differences.

In RASF, the expression of miR-196a is significantly lower than in OASF (p < 0.0001) as well as in RA synovial tissues compared with OA (p = 0.01). RASF transfection with pre-miR/miR-196a inhibitor resulted in down/upregulation of predicted targets HOXC8 and ANXA1. Pre-miR-196a suppressed cell proliferation (27.5%) and migration (41.5%) and induced apoptosis (54.1%) while miR-196a inhibitor enhanced both proliferation (81.9%) and migration (231%) and reduced apoptosis (52.3%) in RASF.

## Conclusion

In contrast to established RA synovial fibroblasts where an increased expression of miR-146a was reported, our data showed that in early arthritis sera miR-146a is significantly downregulated and might characterize an early clinical stage of the disease. The low expression of miR-196a in both RA synovial tissue and in isolated SF contributes to the aggressive and invasive phenotype of RASF by modifying proliferation, migration and apoptosis with an impact on the pathogenesis of RA.
